# Intravenous Therapy Duration and Outcomes in Melioidosis: A New Treatment Paradigm

**DOI:** 10.1371/journal.pntd.0003586

**Published:** 2015-03-26

**Authors:** Matthew C. Pitman, Tara Luck, Catherine S. Marshall, Nicholas M. Anstey, Linda Ward, Bart J. Currie

**Affiliations:** 1 Infectious Diseases Department, Royal Darwin Hospital, Darwin, Northern Territory, Australia; 2 Global and Tropical Health Division, Menzies School of Health Research, Charles Darwin University, Darwin, Northern Territory, Australia; University of California San Diego School of Medicine, UNITED STATES

## Abstract

**Background:**

International melioidosis treatment guidelines recommend a minimum 10 to 14 days’ intravenous antibiotic therapy (intensive phase), followed by 3 to 6 months’ oral therapy (eradication phase). This approach is associated with rates of relapse, defined as recurrence following the eradication phase, that can exceed 5%. Rates of recrudescence, defined as recurrence during the eradication phase, have not previously been reported. In response to low eradication phase completion rates in Australia, a local guideline has evolved over the last ten years recommending a longer minimum intensive phase duration for many cases of melioidosis.

**Methodology/ Principal Findings:**

This retrospective cohort study reviews antibiotic duration for the first episode of care for all patients diagnosed with melioidosis and surviving the intensive phase during a recent three year period in the tropical north of Australia’s Northern Territory; we also review adherence to the current local guideline and treatment outcomes. Of 215 first episodes of melioidosis surviving the intensive phase, the median (interquartile range) intensive phase duration was 26 (14-34) days. One hundred and eight (50.2%) patients completed eradication therapy; 58 (27.0%) patients took no eradication therapy. At 28 months’ follow-up, one (0.5%) relapse and eleven (5.1%) recrudescences had occurred. On exact logistic regression analysis, the only independent risk factors for recrudescence were self-discharge during the intensive phase (odds ratio 6.2 [95% confidence interval 1.2-30.0]) and septic shock (odds ratio 5.3 [95% confidence interval 1.1-25.7]).

**Conclusions/ Significance:**

Relapsed melioidosis is rare in patients who receive a minimum intensive phase duration specified by our guideline and extended according to clinical progress. Recrudescence rates may improve with reductions in rates of self-discharge. Given the low relapse rate despite a high rate of eradication therapy non-adherence, the duration and necessity of eradication therapy for different patients after guideline-concordant intensive therapy should be evaluated further.

## Author Summary

Melioidosis is an infection caused by the soil bacterium *Burkholderia pseudomallei*; patients usually present with pneumonia, blood-stream infection and/or skin or internal organ abscesses. Melioidosis occurs most commonly in northern Australia and parts of Southeast Asia. It has a high mortality rate and, with standard treatment, a relapse rate greater than 5%; patients who relapse often represent severely unwell. Treatment comprises an intensive (intravenous antibiotic) phase, followed by a prolonged eradication (oral antibiotic) phase. Previous studies have found that the intensive phase is important to prevent mortality, and the eradication phase is important to prevent relapse. However, these studies have not been designed to detect an effect of intensive therapy on relapse rate. We know that adherence to eradication therapy is poor, and many of our patients live remotely making follow-up difficult. In order to address this, we have developed a new treatment guideline which stipulates a longer intensive phase for most patients. We show that adherence to this guideline is associated with very low relapse rates despite poor adherence to eradication therapy. It is possible that for many patients the eradication phase could be shortened or avoided when this intensive phase guideline is followed; this requires further research.

## Introduction

*Burkholderia pseudomallei*, the cause of melioidosis, is a soil- and water-borne bacterium endemic to northern Australia and parts of south-east Asia [1]. It most commonly causes pneumonia, bacteremia without evident focus, deep-seated abscesses and skin infection but can affect almost any part of the body [2]. It causes more serious disease in patients with impaired innate immunity such as those with diabetes and has a high mortality rate ranging from 9% to 49% [3–5]. It is intrinsically resistant to most antibiotics and requires prolonged therapy for cure [5–8].

Current international guidelines suggest treatment with a minimum 10 to 14 days’ intravenous antibiotics (intensive phase) followed by 3 to 6 months’ oral antibiotics (eradication phase) [1,9,10]. Options for the intensive phase include ceftazidime or a carbapenem [10–13]; options for the eradication phase include trimethoprim-sulfamethoxazole (TMP-SMX), doxycycline or amoxicillin-clavulanate [10,14–19]. Whilst there is provision in these international guidelines to extend the intensive phase to four weeks or greater in severe cases of melioidosis, the focus of the international guidelines is on switching to the eradication phase once the patient has been afebrile for 48 hours with negative blood cultures and an ability to take medication orally [9,10,17].

Despite treatment according to such guidelines, patients still have a high rate of relapse. In the Northern Territory (NT) of Australia, from 1989 to 2009, 5.2% of 465 patients surviving the intensive phase have had molecularly confirmed relapse [4,20]. In Thailand, rates of relapse between 1986 and 2004 were at least 9.3% [5]. More recent data from a Thai randomized controlled trial demonstrated a relapse rate of somewhere between 1.1% and 6.4% [17]. Choice and duration of oral eradication therapy have been found to be the strongest risk factors for relapse [5].

A substantial proportion of our patients fail to complete the eradication phase and many live in remote communities making follow up difficult. In response to this, intensive phase therapy in our region has been progressively lengthened over the last 10 years. This is reflected in a local guideline developed at Royal Darwin Hospital (RDH) directing duration of therapy according to site of infection which in many cases extends intensive therapy far beyond defervescence with negative blood cultures and an ability to take oral antibiotics. In this study, we review antibiotic duration received by patients with melioidosis over a recent three year period, clinician and patient adherence to the local guideline and associated outcomes.

## Methods

### Design

This study was a retrospective analysis of data from the ongoing Darwin Prospective Melioidosis Study [4,21], a prospective cohort study. The study size was determined by the number of patients diagnosed with melioidosis during a recent period in and since which the guideline being evaluated has remained substantially unchanged.

### Patients

All patients with culture-confirmed melioidosis in the tropical Top End of the NT diagnosed between 1^st^ October 2009 and 30^th^ September 2012 who survived the intensive phase were eligible for inclusion in the study. Antibiotic therapy was managed by the Infectious Diseases Department at RDH. Exclusion criteria included cases that represented recrudescence or relapse of melioidosis first diagnosed prior to 1^st^ October 2009 and cases with incomplete or inaccessible records. Antibiotic type and duration were reviewed for both the intensive and eradication phases along with demographic, clinical and laboratory data; these data had been recorded prospectively as part of the ongoing Darwin Prospective Melioidosis Study [4,21].

### Definitions

Intensive phase was defined as the period of time during which the patient received intravenous therapy directed against *B*. *pseudomallei* irrespective of clinician-intended duration. Eradication phase was defined as the period of time commencing at the end of the intensive phase and finishing at the guideline-recommended end date of eradication therapy irrespective of actual duration received. Intensive and eradication therapy referred to the intravenous and oral antibiotics directed against *B*. *pseudomallei* during the intensive and eradication phase respectively.

Recrudescence and recurrence were defined as return of clinical illness during and after the eradication phase respectively with concomitant culture of *B*. *pseudomallei* from a clinical specimen. Recurrence was further defined as either relapse or reinfection when isolates from the initial and subsequent illness were identical or different respectively by multilocus sequence typing (MLST) [20]. Cure was defined as the absence of death during the eradication phase or recrudescence or relapse at the end of the follow-up period.

Risk factors were defined as in the Darwin Prospective Melioidosis Study [4]. Antibiotic duration-determining focus (ADDF) was the focus requiring the longest minimum intensive phase duration according to the local guideline; if there were two or more such foci requiring the same minimum intensive phase duration, the ADDF was whichever of these appeared lowest on Table 1, this being the focus generally considered most difficult to cure. Self-discharge was defined as voluntary cessation of inpatient status prior to completion of the clinician-planned intensive phase irrespective of guideline minimum duration. Non-adherence to eradication therapy was defined as cessation of eradication therapy prior to recrudescence or, if patients did not recrudesce, prior to the end of the eradication phase.

**Table 1:** Darwin melioidosis guideline.

Antibiotic Duration-Determining Focus		Minimum intensive phase duration (weeks)^a^	Eradication phase duration (days)
Skin abscess		2	90
Bacteremia with no focus		2	90
Pneumonia	without lymphadenopathy^b^ or ICU admission	2	90
	with either lymphadenopathy^b^ or ICU admission	4	90
Deep-seated collection^c^		4^d^	90
Osteomyelitis		6	180
Central nervous system infection		8	180
Arterial infection^e^		8^d^	180

a. Clinical judgement to guide prolongation of intensive phase if improvement is slow or if blood cultures remain positive at 7 days.

b. Defined as enlargement of any hilar or mediastinal lymph node to greater than 10mm diameter.

c. Defined as abscess anywhere other than skin, lungs, bone, CNS or vasculature; septic arthritis is considered a deep-seated collection.

d. Intensive phase duration is timed from date of most recent drainage or resection where culture of the drainage specimen or resected material grew *B*. *pseudomallei* or where no specimen was sent for culture; clock is not reset if specimen is culture-negative. On 1^st^ October 2010, the minimum duration for deep-seated collection changed from 2 to 4 weeks from last such drainage/resection.

e. Most commonly presenting as mycotic aneurysm.

### Guideline

The Darwin Melioidosis Guideline is summarized in Table 1. First line intensive therapy was ceftazidime unless the patient was in the intensive care unit (ICU) or allergic or intolerant to ceftazidime in which case meropenem was used. If there were a collection (including skin abscess or septic arthritis), bone or central nervous system (CNS) involvement, TMP-SMX, doxycycline or amoxicillin-clavulanate (in order of preference) was added early during the intensive phase for tissue penetration, usually orally. Subsequent oral eradication therapy used the same choice of these latter three antibiotics. Eradication phase was 90 days for each ADDF except osteomyelitis, CNS infection and arterial infection when 180 days was used. As noted in Table 1, there was a small change to the guideline on 1^st^ October 2010; clinician adherence was assessed against the final version of the guideline.

### Follow-up

Patients who survived the intensive phase were generally reviewed at monthly infectious diseases outpatient visits until completion of eradication therapy. Where patients failed to attend appointments, eradication therapy was assumed to have ceased at the last infectious diseases appointment attended or the last of any subsequent entries documenting therapy in the RDH medical record or NT-wide shared electronic health record. Follow-up data until 1^st^ December 2014 were included; follow-up between the last clinic appointment and 1^st^ December 2014 was performed retrospectively by reviewing hospital and community shared electronic health records. Melioidosis is a notifiable disease in the NT; data on melioidosis recurrence were based on Australia-wide laboratory notification of positive cultures to the NT public health unit.

### Data analysis

Data not normally distributed were expressed as median ± interquartile range (IQR). Bivariate analysis of categorical and continuous variables was performed using the two-tailed Fisher exact test (due to low expected cell values) and the Wilcoxon rank-sum test respectively. Significant variables on bivariate analysis at p < 0.05 were assessed by multivariate analysis using exact logistic regression; exact methods were used due to the infrequency of recrudescence. Stepwise elimination of variables least significant on bivariate analysis was performed until all variables remaining in the model were statistically significant. Analyses were performed using Stata version 12 (StataCorp, College Station, TX).

### Ethics

This study was approved by the Human Research Ethics Committee of the NT Department of Health and the Menzies School of Health Research (HREC 02/38). As this was a retrospective observational study of a large dataset of a notifiable disease and data were analyzed anonymously, consent was not required.

## Results

### Patients

Two hundred and fifty patients were diagnosed with melioidosis and managed by the RDH Infectious Diseases Department between 1^st^ October 2009 and 30^th^ September 2012. Twenty-seven (10.8%) of these died during the intensive phase, one was a relapse of a case diagnosed prior to 1^st^ October 2009 and a further seven had incomplete antibiotic data due to missing files or partially interstate treatment; these patients were excluded leaving 215 patients in the study. All 215 patients were followed up as outlined in the methods and data analysis was performed on all patients except where stated.

Baseline characteristics are shown in Table 2. The median (IQR) age was 49.6 (39.0–60.5) years; 119 (55.3%) patients were male and 15 (7.0%) were under 18 years of age. The cohort had a high rate of comorbidity with 181 (84.2%) patients having at least one recognized risk factor for melioidosis. In addition, melioidosis was of characteristic severity with 128 (59.5%) patients bacteremic, 47 (21.9%) requiring intensive care and 32 (14.9%) developing septic shock.

**Table 2:** Baseline characteristics.

Baseline characteristic		Number (%^a^) except where indicated
Demographics	Male	119 (55.3)
	Age	49.6 (39.0–60.5)^b^
Risk factor	Any risk	181 (84.2)
	Diabetes	100 (46.5)
	Hazardous alcohol use	86 (40.0)
	Chronic lung disease	59 (27.4)
	Chronic renal disease	27 (12.6)
	Malignancy	26 (12.1)
	Immunosuppression	26 (12.1)
	Congestive cardiac failure / rheumatic heart disease	21 (9.8)
Severity	Bacteremia	128 (59.5)
	ICU admission	47 (21.9)
	Septic shock	32 (14.9)
Antibiotic duration-determining focusDeep-seated collection	Skin abscess	26 (12.1)
	Bacteremia no focus	14 (6.5)
	Pneumonia	102 (47.4)
	Deep-seated collection	56 (26.0)
	Osteomyelitis	12 (5.6)
	Central nervous system infection	4 (1.9)
	Arterial infection	1 (0.5)
Deep-seated collection	Prostate	23 (10.7)
	Septic arthritis	13 (6.0)
	Spleen	12 (5.6)
	Liver	10 (4.7)
	Deep soft tissue	9 (4.2)
	Kidney	7 (3.3)
	Muscle	6 (2.8)
	Pericardial	3 (1.4)
	Other	9 (4.2)

a. The denominator for all percentage calculations is 215; there were no missing values.

b. Median (interquartile range).

### Treatment

Intensive therapy duration is shown in Table 3. The median (IQR) intensive phase duration for the 215 patients overall was 26 (14–34) days. Twenty (9.3%) patients self-discharged during the intensive phase. Of 133 (61.9%) patients who completed their intravenous therapy through the RDH Hospital in the Home program, the median (IQR) duration of infusor therapy was 14 (8–22) days.

**Table 3:** Duration of intensive therapy.

Antibiotic		Patients	No. of patients	Duration (days)	
				Median	Interquartile range
Meropenem and ceftazidime	Total	Total	215	26	14–34
		Non-self-dischargers	195	27	14–35
		Self-dischargers	20	17	13–25.5
	From last drainage	Total	215	25	14–30
		Non-self-dischargers	195	27	14–31
		Self-dischargers	20	17	12.75–25.5
Meropenem^a^	Total	Total	97	7	3–18
		Non-self-dischargers	86	7	3–18
		Self-dischargers	11	5	3.5–16
	From last drainage	Total	97	6	2–16
		Non-self-dischargers	86	6	2–16
		Self-dischargers	11	5	3–16
Ceftazidime^b^	Total	Total	204	18.5	14–28.25
		Non-self-dischargers	185	19	14–29
		Self-dischargers	19	13	10.5–22
	From last drainage	Total	204	17	14–28
		Non-self-dischargers	185	18	14–28
		Self-dischargers	19	13	10.5–22

a. Excludes 118 patients who did not receive any meropenem.

b. Excludes 11 patients who did not receive any ceftazidime.

**Fig 1:**
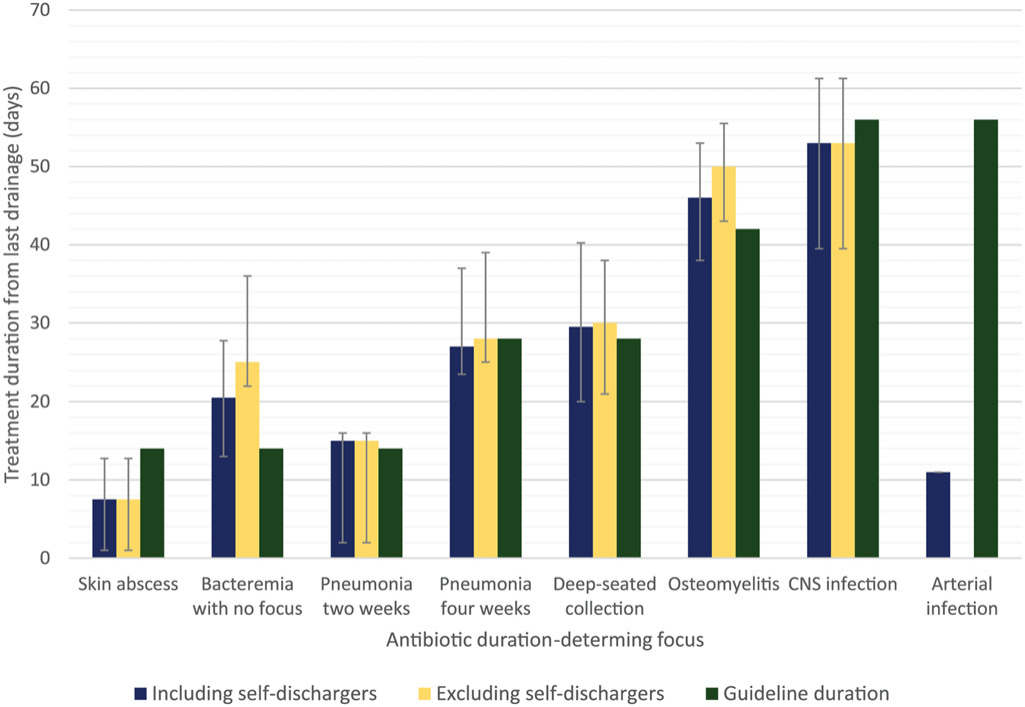
Median treatment duration from last drainage ± interquartile range in comparison to minimum guideline duration.

**Fig 2:**
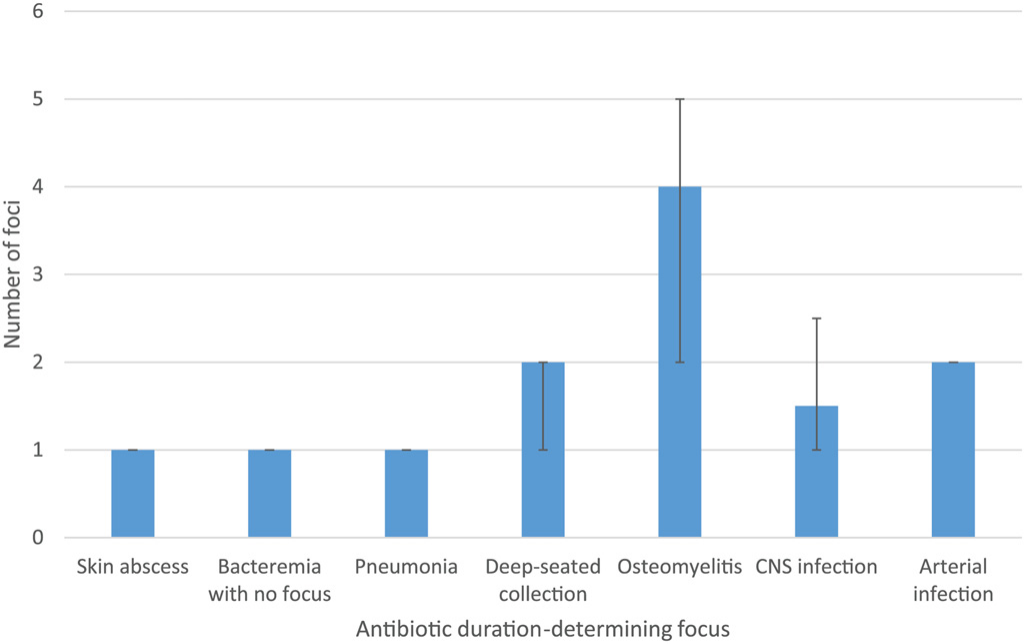
Median number of foci of infection ± interquartile range according to antibiotic duration-determining focus.

Median intensive phase duration according to ADDF is shown in Fig. 1. When self-dischargers were excluded, patients with bacteremia with no focus or osteomyelitis as an ADDF tended to have a longer intensive phase duration than the guideline minimum duration. For patients with bacteremia with no focus, the most common reasons for prolonged therapy were immunosuppression (1 on cancer chemotherapy, 1 on high dose dexamethasone for cerebral metastases and 1 with systemic lupus erythematosus-related neutropenia) and, in hindsight, incorrectly suspected other foci of infection (3 patients). Patients with osteomyelitis were more likely to have multifocal disease as depicted in Fig. 2 and tended to be slower to improve resulting in an extension of the intensive phase.

Eradication phase duration was bimodal with one peak occurring at 90 days (93 [43.3%] patients) and a second peak at 0 days (58 [27.0%] patients). Only 108 (50.2%) patients completed the guideline-specified eradication phase. In total, 70.7% of eradication therapy days were with TMP-SMX; 29.3% were with doxycycline. No patients received amoxicillin-clavulanate.

### Clinician adherence to intensive phase duration

**Fig 3:**
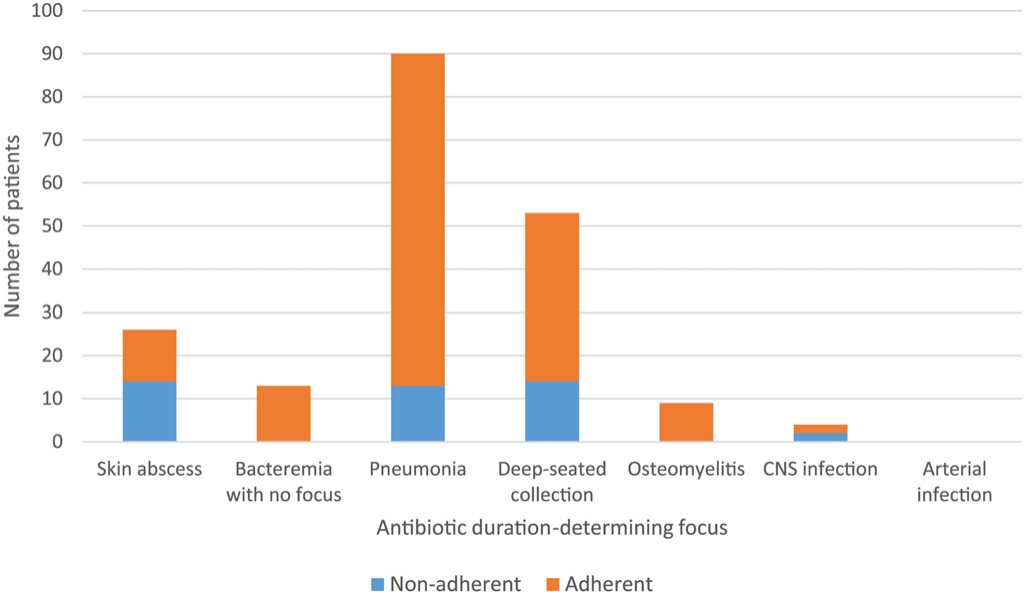
Clinician adherence to guideline-specified minimum intensive phase duration excluding patients who self-discharged.

Adherence to the guideline-specified minimum intensive phase duration by clinicians according to ADDF is demonstrated in Fig. 3; self-discharging patients are excluded as it was not possible for clinicians to adhere in these cases. Of the remaining 195 patients, 43 (22.1%) received less than the guideline-directed minimum intensive phase duration. In most cases of clinician non-adherence, the ADDF was skin abscess, pneumonia or deep-seated collection. Of the pneumonia cases, most had minor degrees of hilar/mediastinal lymphadenopathy and did not receive four weeks’ intensive therapy; of the deep-seated collection cases, most occurred in the first year of the study when it was common for patients to receive two rather than four weeks’ intensive therapy from last drainage. Despite this, 42/43 (97.7%) clinician non-adherent cases had cure of their infection.

### Outcomes

Of the 215 patients, 197 (91.6%) patients were cured. The median (range) duration of follow-up from the onset of the eradication phase for these cured patients was 45.9 (28.4–61.1) months.

Six (2.8%) patients died during the eradication phase. All six were palliated with eradication therapy ceased by the attending physicians prior to death. Five patients had incurable malignancy and one had advanced dementia. Two of the six patients had fever before dying but were not investigated due to their palliative status. A third patient, a 64 year old man with bacteremia with no focus, had been treated with four weeks’ ceftazidime followed by two weeks’ cotrimoxazole; this was then ceased due to a rash and was not replaced due to his palliative status in the context of squamous cell carcinoma of the lung with brain metastases, hypercalcemia and delirium requiring dexamethasone. He died 27 days after cessation of cotrimoxazole. Three days prior to dying he had a throat swab collected to investigate pneumonia; this grew *B*. *pseudomallei* 2 days after death. He was counted as a death during the eradication phase rather than a recrudescence as he was being actively palliated. The remainder of the six patients had no clinical evidence of infection prior to death.

Eleven (5.1%) patients recrudesced after their initial admission. The median (IQR) time to recrudescence from the end of the intensive phase was 24 (13–43) days; the median (IQR) time from the last day of taking eradication therapy was 10 (0–22) days with four (36%) patients still taking their eradication therapy when they recrudesced. Of these four, three had an appropriate intensive phase duration according to the guideline but two of these recrudesced with infected foreign bodies that had not initially been evident clinically (staghorn calculus and suspected vascular graft infection) and the third had bronchiectasis with persistent sputum positivity.

Only one (0.5%) relapse occurred. This was a 34 year old man with pneumonia, mediastinal lymphadenopathy and an undrained liver abscess who self-discharged after 15 days’ intensive therapy and took no eradication therapy. He relapsed 15 months later with severe sepsis due to worsening pulmonary abscesses and mediastinal lymphadenopathy but had a stable-appearing liver abscess.

One (0.5%) patient had reinfection. He was considered for the purpose of analysis to have had cure of his original infection.

There were no patients with clinically-suspected but culture-unproven recrudescence or recurrence.

Bivariate analysis was performed to assess for significant predictors of recrudescence. Patients who died during the eradication phase were excluded due to a shorter exposure to the possibility of recrudescence and a likely bias toward not diagnosing recrudescence due to the palliative nature of these patients. This left 209 patients in the analysis. Results of analysis of categorical variables are shown in Table 4. The median (IQR) age for patients who did and did not recrudesce was 46.3 (41.2 to 55.6) and 49.6 (38.8–59.9) years respectively (p = 0.47). The only variables significantly predicting recrudescence were diabetes, having osteomyelitis as an ADDF, admission to ICU, septic shock and self-discharge. Multivariate analysis showed that only two of these variables were statistically significant independent predictors of recrudescence; these were self-discharge (odds ratio 6.2 [95% confidence interval 1.2–30.0, p < 0.05]) and septic shock (odds ratio 5.3 [95% confidence interval 1.1–25.7, p < 0.05]).

**Table 4:** Bivariate analysis of categorical risk factors for recrudescence.

Risk factor		Number (%^a^)	Odds ratio (95% confidence interval)	P value
Demographics	Male	115 (55.0)	0.98 (0.24–4.20)	1.000
Risk factor	Any risk	175 (83.7)	undefined	0.218
	Diabetes	97 (46.4)	5.63 (1.12–54.41)*	0.026
	Hazardous alcohol use	86 (41.1)	1.20 (0.28–4.91)	0.763
	Chronic lung disease	56 (26.8)	1.60 (0.33–6.60)	0.490
	Chronic renal disease	26 (12.4)	0.69 (0.02–5.26)	1.000
	Immunosuppression	23 (11.0)	0.00 (0.00–2.74)	0.615
	Malignancy	21 (10.0)	0.89 (0.02–6.88)	1.000
	Congestive cardiac failure / rheumatic heart disease	21 (10.0)	0.00 (0.00–3.05)	0.607
Severity	Bacteremia	122 (58.4)	1.96 (0.45–11.80)	0.367
	ICU admission	47 (22.5)	6.91 (1.64–33.40)**	0.003
	Septic shock	31 (14.8)	8.30 (1.92–36.55)**	0.002
ADDF	Skin abscess	26 (12.4)	0.00 (0.00–2.38)	0.366
	Bacteremia no focus	13 (6.2)	0.00 (0.00–5.22)	1.000
	Pneumonia	99 (47.4)	0.62 (0.13–2.53)	0.544
	Deep-seated collection	54 (25.8)	1.08 (0.18–4.72)	1.000
	Osteomyelitis	12 (5.7)	7.88 (1.13–40.2)*	0.019
	Central nervous system infection	4 (1.9)	0.00 (0.00–18.46)	1.000
Adequacy of treatment	Self-discharge	20 (9.6)	10.17 (2.14–44.52)**	0.002
	≤ 2 weeks intensive phase	61 (29.2)	0.52 (0.05–2.65)	0.515
	Less than minimum intensive phase per guideline	81 (38.8)	2.93 (0.71–14.06)	0.112
	Non-adherence to eradication therapy	97 (46.4)	2.10 (0.51–10.07)	0.353
	Doxycycline vs cotrimoxazole as predominant eradication therapy	41 (26.8)^b^	2.92 (0.512–16.38)	0.211
	No eradication therapy vs cotrimoxazole as predominant eradication therapy	56 (33.3)^c^	1.53 (0.216–9.36)	0.687

a. The denominator for all percentage calculations is 209 except where stated; there were no missing values.

b. Numerator and denominator for percentage calculation represent number of patients with doxycycline and either doxycycline or cotrimoxazole as the predominant eradication therapy respectively.

c. Numerator and denominator for percentage calculation represent number of patients with no eradication therapy and either no eradication therapy or cotrimoxazole as the predominant eradication therapy respectively.

A subgroup analysis was performed on the 58 patients who took no eradication therapy; 52 (89.7%) were cured, 2 (3.4%) died during the eradication phase, 3 (5.2%) recrudesced and 1 (1.7%) relapsed.

## Discussion

The evolving therapy of melioidosis over the last 25 years has largely been informed by sequential randomized controlled trials from Thailand [11,13–17,22–27]. All trials assessing intensive phase therapy in melioidosis have had mortality as a primary endpoint; none has included relapse as an endpoint [11,13,22,23,25–27]. Minimum duration of intensive therapy in these studies has been 7–14 days with a median of 7–15 days’ therapy actually administered. No study has randomized patients to different intensive phase durations. All randomized controlled trials assessing eradication therapy have had relapse as a primary endpoint [14–17,24]; these studies have repeatedly found that eradication therapy choice, duration and compliance affect relapse rate whereas intensive phase choice and duration do not.

Based on these studies, current international guidelines recommend a minimum of 10 days’ intravenous therapy for melioidosis [1,9]. However, patients treated according to these guidelines still have a high relapse rate. In the most comprehensive retrospective study of relapse [5], isolates were analyzed for all 921 patients diagnosed from 1986 to 2004 in northeast Thailand who survived the intensive phase and presented for follow-up; 9.3% of patients had a proven relapse although a further 3.5% did not have paired isolates available for testing [5]. Not all patients in this study had been treated according to current guidelines as many had been part of randomized controlled trials evaluating experimental treatment regimens; indeed, only in 1994 was the minimum intensive phase duration switched from 7 to 10 days. Nonetheless, choice and duration of eradication therapy were the strongest predictors of relapse; other risk factors included bacteremia and multifocal disease. Choice and duration of intravenous therapy were found not to be risk factors for relapse [5].

This Thai study defined relapse as new symptoms and signs of infection plus *B*. *pseudomallei* culture positivity after an initial response to therapy [5], similar to a combination of our definitions for recrudescence and relapse. Given that the median (IQR) time to proven relapse in the Thai study was 26 (10–72) weeks and 62% of patients had more than 16 weeks’ eradication therapy [5], it is likely that a proportion of such relapses were actually recrudescences according to our definition.

In a recent Thai randomized controlled trial [17], which followed the current international guidelines, the rate of culture-confirmed melioidosis recurrence was 5.9% but it was not possible to determine what proportion of these were recrudescences, relapses or reinfections according to our definition. Based on the data provided, the maximum possible range for the relapse rate was 1.1–6.4%; this range includes patients with clinically suspected but culture-unproven relapse (maximum 2.9%). Inclusion criteria for this trial included satisfactory completion of intensive therapy and a high likelihood of completing at least six months’ follow-up; thus this relapse rate may not be externally valid for the general population.

The relapse rate in the NT from 1989 to 2009, defined as recurrence with the same MLST occurring after the end of the eradication phase, was 5.2% [4,20]. This rate has decreased over the last decade in parallel with a progressive lengthening of the intensive phase to compensate for poor eradication therapy adherence [20]. The current guideline is associated with a median 26 day intensive phase duration. That only 1/215 (0.5%) patients relapsed and yet only 50.2% of patients completed the eradication phase suggests that the longer duration of the intensive phase is important in preventing relapse. It is possible that further relapses could occur after the follow-up period but, considering that our shortest follow-up duration, 28.4 months, well exceeds the median time to relapse in the NT, 8 months [4], most relapses would be expected to occur within the follow-up period.

That 42 of 43 (97.7%) cases where clinicians were not adherent to the local guideline were cured suggests that the guideline can be further refined. For instance, it is common in the NT to give only two or three days’ intensive therapy for skin abscesses pending exclusion of other foci on culture and imaging and it may be that no intensive therapy is necessary if adherence to oral therapy is likely in these patients. Oral therapy alone in some cases of skin abscess has been successful in Australia [28,29], Thailand [30] and Cambodia [31] but careful exclusion of other foci of infection and a high likelihood of oral therapy adherence are necessary if initial intravenous therapy is to be omitted. The importance of extending intensive therapy for pneumonia to four weeks for minor degrees of mediastinal or hilar lymphadenopathy is unclear; however, in many countries where melioidosis occurs, x-rays are the only form of chest imaging available and these will only detect gross lymphadenopathy. While lymphadenopathy may reflect inhalational melioidosis and therefore potential for more severe disease [32], concerns about radiation dose with computed tomography (CT) scanning caution against routine CT imaging of the chest [33]. CT scans of the chest were not performed routinely in our study; however, our analysis did include patients whose lymphadenopathy was only detected on CT chest. In the case of deep-seated collection, it may be appropriate to refine the current guideline to a minimum two weeks’ intensive therapy from last culture-positive drainage provided there is a minimum of four weeks’ intensive therapy in total.

Whilst the recrudescence rate was higher than desired at 5.1%, there are no previous data, including from our region, on strictly-defined recrudescence with which to compare our current data; recrudescence in the Darwin Prospective Melioidosis Study has traditionally been considered as part of the primary episode of infection while in the Thai studies it has sometimes been included in the relapse data. Of note, the 5.2% historical relapse rate from our region [4] used the same definition of relapse reported here and therefore did not include any recrudescences; when recrudescences occurred, they were considered part of the primary episode of infection. That recrudescences occurred in the current study supports that our guideline minimum intensive phase durations were overall not too long.

Many of the cases of recrudescence were explainable. That self-discharge was a significant independent risk factor for recrudescence suggests that if fewer patients had self-discharged we would likely have observed a lower recrudescence rate. Ongoing efforts are required to engage these often highly vulnerable patients in inpatient care. Septic shock as an independent risk factor may reflect a higher initial bacterial burden and degree of immune suppression and thus a requirement for a longer intensive phase to eradicate infection. This underscores the importance of extension of intensive therapy beyond the guideline minimum duration if clinical improvement is slow. As noted, there were two additional observed causes for recrudescence in this study which were not tested in the analysis due to inadequate numbers, these being infected foreign body (two patients) [5,34,35] and bronchiectasis (one patient) [36–38].

Whilst there was a correlation between self-discharge and eradication therapy non-adherence, in that all 20 patients who self-discharged were non-adherent, these patients accounted for only 20.6% of the 97 patients who were non-adherent. This explains the difference in outcome on bivariate analysis for these two variables.

The discrepancy between our finding that eradication therapy adherence was not important in predicting recrudescence or relapse and the Thai finding that choice and duration of eradication therapy were the most important predictors of relapse [5] may reflect that most of our patients were already cured by the longer intensive phase whereas the Thai patients were more dependent on the eradication phase after a generally shorter intensive phase. The lack of a significant effect of duration of intensive phase on relapse rate in the Thai studies may reflect inadequate diversity in intensive phase duration to detect such an effect [5,15].

The strengths of our study are the number of patients included and that most data were collected prospectively. One limitation is that much follow-up data was collected retrospectively. However, the NT has a robust shared electronic health record facilitated by an NT-wide unique patient identification number allowing confirmation of outcome. Additionally, as melioidosis is a notifiable disease in the NT, all Australian laboratories are mandated to report positive cultures of NT patients to the NT public health unit which liaises with the RDH Infectious Diseases Department. We acknowledge that there remains a theoretical possibility that patients with recrudescent or recurrent melioidosis diagnosed after migration overseas may have been missed.

Another limitation is that results are compared with historical findings [4] and attribution of improvement in relapse rate to the guideline may be confounded by other interventions. Furthermore, as the guideline evolved over a ten year period, it was not possible to use historical controls from a short period immediately preceding the period studied. Nonetheless, we are not aware of any other changes in management which may have confounded outcomes. There is biological plausibility in attributing lower relapse rates to the guideline in that intensive therapy uses potent bactericidal antibiotics whereas eradication therapy uses bacteriostatic antibiotics. There has been a significant improvement in the melioidosis mortality rate over the last twenty years in our region, attributed largely to improved intensive care [4], which has meant that more patients with septic shock are surviving; these patients are at higher risk of recrudescence and relapse [5] and yet they are not relapsing. Based on the above data and decades of clinical experience, we do not believe that there is equipoise in our region for a randomized controlled trial comparing traditional two weeks’ intensive therapy with our guideline minimum intensive phase duration.

Our findings have important implications for management of melioidosis globally. Relapse rates with traditional approaches to melioidosis therapy are high and present a significant risk to patients and a burden on healthcare resources. Adoption of our guidelines may significantly reduce relapse rates; however, many hospitals in Southeast Asia do not have Hospital in the Home programs to facilitate domiciliary intravenous antibiotic administration and the cost of intravenous ceftazidime itself is often prohibitive. Thus, significant improvements in funding and resources may be required before our guideline can be adopted in these settings. Given the near-zero relapse rate in our study, despite poor adherence to eradication therapy, further research is required to evaluate eradication phase duration and necessity for different ADDFs following guideline-concordant therapy. If abbreviating or ceasing therapy after the intensive phase were associated with no excess relapses, this would significantly shorten and improve tolerability and safety of the overall treatment regimen and obviate the need for long term follow up, thus further improving the cost-benefit ratio of our approach.

### Conclusions

We have developed a guideline for duration of intensive phase therapy for melioidosis that we think is responsible for the very low rate of relapse now seen in the Top End of the NT. Its immediate applicability in some developing regions is uncertain due to funding and resource issues. However, given the excellent cure rates with our intensive phase guideline despite poor adherence to subsequent eradication therapy, we believe that further research evaluating the duration and necessity of the eradication phase for different ADDFs is now warranted.

## Supporting Information

S1 Checklist STROBE checklist. (DOCX)
