# Correction: Intravenous Therapy Duration and Outcomes in Melioidosis: A New Treatment Paradigm

**DOI:** 10.1371/journal.pntd.0003737

**Published:** 2015-04-22

**Authors:** The PLOS Neglected Tropical Diseases Staff

In Table 2, there is an error that causes the fourth heading in the left-hand column to be incorrect. The correct text is: Antibiotic duration-determining focus. Please see the corrected Table 2 here.

**Table 2:** Baseline characteristics

Baseline characteristic		Number (%^a^) except where indicated
Demographics	Male	119 (55.3)
	Age	49.6 (39.0–60.5)^b^
Risk factor	Any risk	181 (84.2)
	Diabetes	100 (46.5)
	Hazardous alcohol use	86 (40.0)
	Chronic lung disease	59 (27.4)
	Chronic renal disease	27 (12.6)
	Malignancy	26 (12.1)
	Immunosuppression	26 (12.1)
	Congestive cardiac failure / rheumatic heart disease	21 (9.8)
Severity	Bacteremia	128 (59.5)
	ICU admission	47 (21.9)
	Septic shock	32 (14.9)
Antibiotic duration-determining focus	Skin abscess	26 (12.1)
	Bacteremia no focus	14 (6.5)
	Pneumonia	102 (47.4)
	Deep-seated collection	56 (26.0)
	Osteomyelitis	12 (5.6)
	Central nervous system infection	4 (1.9)
	Arterial infection	1 (0.5)
Deep-seated collection	Prostate	23 (10.7)
	Septic arthritis	13 (6.0)
	Spleen	12 (5.6)
	Liver	10 (4.7)
	Deep soft tissue	9 (4.2)
	Kidney	7 (3.3)
	Muscle	6 (2.8)
	Pericardial	3 (1.4)
	Other	9 (4.2)

^a^The denominator for all percentage calculations is 215; there were no missing values.

^b^Median (interquartile range).
