# Supplementary material for: Intravenous Therapy Duration and Outcomes in Melioidosis: A New Treatment Paradigm
Source: PLoS Negl Trop Dis. 2015 Mar 26;9(3):e0003586. doi: 10.1371/journal.pntd.0003586 (PMC4374799; doi:10.1371/journal.pntd.0003586)
Supplement: S1 Checklist — (DOCX) [file pntd.0003586.s001.docx]

STROBE Statement—checklist of items that should be included in reports of observational studies

|  | Item No. | Recommendation | Page  No. | Relevant text from manuscript |
| --- | --- | --- | --- | --- |
| **Title and abstract** | 1 | (*a*) Indicate the study’s design with a commonly used term in the title or the abstract | 2 | Retrospective cohort study |
|  |  | (*b*) Provide in the abstract an informative and balanced summary of what was done and what was found | 2 | This retrospective cohort study reviews antibiotic duration for the first episode of care for all patients diagnosed with melioidosis and surviving the intensive phase during a recent three year period in the tropical north of Australia’s Northern Territory; we also review adherence to the current local guideline and treatment outcomes. Of 215 first episodes of melioidosis surviving the intensive phase, the median (interquartile range) intensive phase duration was 26 (14-34) days. One hundred and eight (50.2%) patients completed eradication therapy; 58 (27.0%) patients took no eradication therapy. At 28 months’ follow-up, one (0.5%) relapse and eleven (5.1%) recrudescences had occurred. On exact logistic regression analysis, the only independent risk factors for recrudescence were self-discharge during the intensive phase (odds ratio 6.2 [95% confidence interval 1.2-30.0]) and septic shock (odds ratio 5.3 [95% confidence interval 1.1-25.7]). |
| Introduction | | | |  |
| Background/rationale | 2 | Explain the scientific background and rationale for the investigation being reported | 4  4  5 | Current international guidelines suggest treatment with a minimum 10 to 14 days’ intravenous antibiotics (intensive phase) followed by 3 to 6 months’ oral antibiotics (eradication phase) [[1](#_ENREF_1),[9](#_ENREF_9),[10](#_ENREF_10)]. … Whilst there is provision in these international guidelines to extend the intensive phase to four weeks or greater in severe cases of melioidosis, the focus of the international guidelines is on switching to the eradication phase once the patient has been afebrile for 48 hours with negative blood cultures and an ability to take medication orally [[9](#_ENREF_9),[10](#_ENREF_10),[17](#_ENREF_17)].  Despite treatment according to such guidelines, patients still have a high rate of relapse. In the Northern Territory (NT) of Australia, from 1989 to 2009, 5.2% of 465 patients surviving the intensive phase have had molecularly confirmed relapse [[4](#_ENREF_4),[20](#_ENREF_20)]. In Thailand, rates of relapse between 1986 and 2004 were at least 9.3% [[5](#_ENREF_5)]. More recent data from a Thai randomized controlled trial demonstrated a relapse rate of somewhere between 1.1% and 6.4% [[17](#_ENREF_17)]. Choice and duration of oral eradication therapy have been found to be the strongest risk factors for relapse [[5](#_ENREF_5)].  A substantial proportion of our patients fail to complete the eradication phase and many live in remote communities making follow up difficult. In response to this, intensive phase therapy in our region has been progressively lengthened over the last 10 years. This is reflected in a local guideline developed at Royal Darwin Hospital (RDH) directing duration of therapy according to site of infection which in many cases extends intensive therapy far beyond defervescence with negative blood cultures and an ability to take oral antibiotics. |
| Objectives | 3 | State specific objectives, including any prespecified hypotheses | 5 | In this study, we review antibiotic duration received by patients with melioidosis over a recent three year period, clinician and patient adherence to the local guideline and associated outcomes. (No pre-specified hypotheses.) |
| Methods | | | |  |
| Study design | 4 | Present key elements of study design early in the paper | 5 | This study was a retrospective analysis of data from the ongoing Darwin Prospective Melioidosis Study [[4](#_ENREF_4),[21](#_ENREF_21)], a prospective cohort study. The study size was determined by the number of patients diagnosed with melioidosis during a recent period in and since which the guideline being evaluated has remained substantially unchanged. |
| Setting | 5 | Describe the setting, locations, and relevant dates, including periods of recruitment, exposure, follow-up, and data collection | 5-6  7 | All patients with culture-confirmed melioidosis in the tropical Top End of the NT diagnosed between 1^st^ October 2009 and 30^th^ September 2012 who survived the intensive phase were eligible for inclusion in the study. Antibiotic type and duration were reviewed for both the intensive and eradication phase along with demographic, clinical and laboratory data; these data had been recorded prospectively as part of the ongoing Darwin Prospective Melioidosis Study [[4](#_ENREF_4),[21](#_ENREF_21)].  Follow-up data until 1^st^ December 2014 were included. |
| Participants | 6 | (*a*) *Cohort study*—Give the eligibility criteria, and the sources and methods of selection of participants. Describe methods of follow-up | 5  7 | All patients with culture-confirmed melioidosis in the tropical Top End of the NT diagnosed between 1^st^ October 2009 and 30^th^ September 2012 who survived the intensive phase were eligible for inclusion in the study…. Exclusion criteria included cases that represented recrudescence or relapse of melioidosis first diagnosed prior to 1^st^ October 2009 and cases with incomplete or inaccessible records.  Patients who survived the intensive phase were generally reviewed at monthly infectious diseases outpatient visits until completion of eradication therapy. Where patients failed to attend appointments, eradication therapy was assumed to have ceased at the last infectious diseases appointment attended or the last of any subsequent entries documenting therapy in the RDH medical record or NT-wide shared electronic health record. Follow-up data until 1^st^ December 2014 were included; follow-up between the last clinic appointment and 1^st^ December 2014 was performed retrospectively by reviewing hospital and community shared electronic health records. Melioidosis is a notifiable disease in the NT; data on melioidosis recurrence were based on Australia-wide laboratory notification of positive cultures to the NT public health unit. |
|  |  | (*b*) *Cohort study*—For matched studies, give matching criteria and number of exposed and unexposed |  | N/A |
| Variables | 7 | Clearly define all outcomes, exposures, predictors, potential confounders, and effect modifiers. Give diagnostic criteria, if applicable | 6  6  6 | Intensive phase was defined as the period of time during which the patient received intravenous therapy directed against *B. pseudomallei* irrespective of clinician-intended duration. Eradication phase was defined as the period of time commencing at the end of the intensive phase and finishing at the guideline-recommended end date of eradication therapy irrespective of actual duration received. Intensive and eradication therapy referred to the intravenous and oral antibiotics directed against *B. pseudomallei* during the intensive and eradication phase respectively.  Recrudescence and recurrence were defined as return of clinical illness during and after the eradication phase respectively with concomitant culture of *B. pseudomallei* from a clinical specimen. Recurrence was further defined as either relapse or reinfection when isolates from the initial and subsequent illness were identical or different respectively by multilocus sequence typing (MLST) [[20](#_ENREF_20)]. Cure was defined as the absence of death during the eradication phase or recrudescence or relapse at the end of the follow-up period.  Risk factors were defined as in the Darwin Prospective Melioidosis Study [[4](#_ENREF_4)]. Antibiotic duration-determining focus (ADDF) was the focus requiring the longest minimum intensive phase duration according to the local guideline; if there were two or more such foci requiring the same minimum intensive phase duration, the ADDF was whichever of these appeared lowest on Table 1, this being the focus generally considered most difficult to cure. Self-discharge was defined as voluntary cessation of inpatient status prior to completion of the clinician-planned intensive phase irrespective of guideline minimum duration. Non-adherence to eradication therapy was defined as cessation of eradication therapy prior to recrudescence or, if patients did not recrudesce, prior to the end of the eradication phase. |
| Data sources/ measurement | 8* | For each variable of interest, give sources of data and details of methods of assessment (measurement). Describe comparability of assessment methods if there is more than one group | 5-6  7 | Antibiotic type and duration were reviewed for both the intensive and eradication phases along with demographic, clinical and laboratory data; these data had been recorded prospectively as part of the ongoing Darwin Prospective Melioidosis Study [[4](#_ENREF_4),[21](#_ENREF_21)].  Where patients failed to attend appointments, eradication therapy was assumed to have ceased at the last infectious diseases appointment attended or the last of any subsequent entries documenting therapy in the RDH medical record or NT-wide shared electronic health record. Follow-up data until 1^st^ December 2014 were included; follow-up between the last clinic appointment and 1^st^ December 2014 was performed retrospectively by reviewing hospital and community shared electronic health records. Melioidosis is a notifiable disease in the NT; data on melioidosis recurrence were based on Australia-wide laboratory notification of positive cultures to the NT public health unit. |
| Bias | 9 | Describe any efforts to address potential sources of bias | 16 | One limitation is that much follow-up data was collected retrospectively. However, the NT has a robust shared electronic health record facilitated by an NT-wide unique patient identification number allowing confirmation of outcome. Additionally, as melioidosis is a notifiable disease in the NT, all Australian laboratories are mandated to report positive cultures of NT patients to the NT public health unit which liaises with the RDH Infectious Diseases Department. We acknowledge that there remains a theoretical possibility that patients with recrudescent or recurrent melioidosis diagnosed after migration overseas may have been missed. |
| Study size | 10 | Explain how the study size was arrived at | 5 | The study size was determined by the number of patients diagnosed with melioidosis during a recent period in and since which the guideline being evaluated has remained substantially unchanged. |

Continued on next page

| Quantitative variables | 11 | Explain how quantitative variables were handled in the analyses. If applicable, describe which groupings were chosen and why | 6  27  31 | Risk factors were defined as in the Darwin Prospective Melioidosis Study [[4](#_ENREF_4)].... Self-discharge was defined as voluntary cessation of inpatient status prior to completion of the clinician-planned intensive phase irrespective of guideline minimum duration. Non-adherence to eradication therapy was defined as cessation of eradication therapy prior to recrudescence or, if patients did not recrudesce, prior to the end of the eradication phase.  Table 2 (for continuous vs categorical variables; also for numbers of cases and percentage of total at risk for each variable)  Table 4 (for continuous vs categorical variables; also for numbers of cases and percentage of total at risk for each variable) |
| --- | --- | --- | --- | --- |
| Statistical methods | 12 | (*a*) Describe all statistical methods, including those used to control for confounding | 8 | Data not normally distributed were expressed as median ± interquartile range (IQR). Bivariate analysis of categorical and continuous variables was performed using the two-tailed Fisher exact test (due to low expected cell values) and the Wilcoxon rank-sum test respectively. Significant variables on bivariate analysis at p < 0.05 were assessed by multivariate analysis using exact logistic regression; exact methods were used due to the infrequency of recrudescence. Stepwise elimination of variables least significant on bivariate analysis was performed until all variables remaining in the model were statistically significant. Analyses were performed using Stata version 12 (StataCorp, College Station, TX). |
|  |  | (*b*) Describe any methods used to examine subgroups and interactions | 12 | A subgroup analysis was performed on the 58 patients who took no eradication therapy; 52 (89.7%) were cured, 2 (3.4%) died during the eradication phase, 3 (5.2%) recrudesced and 1 (1.7%) relapsed. |
|  |  | (*c*) Explain how missing data were addressed | 28  32 | There were no missing values.  There were no missing values. |
|  |  | (*d*) *Cohort study*—If applicable, explain how loss to follow-up was addressed | 8-9 | All 215 patients were followed up as outlined in the methods  [ie not applicable – the main outcome was determined by laboratory notification to the Northern Territory public health unit which is considered to be a complete data set.] |
|  |  | (*e*) Describe any sensitivity analyses |  | N/A |
| Results | | | | |
| Participants | 13* | (a) Report numbers of individuals at each stage of study—eg numbers potentially eligible, examined for eligibility, confirmed eligible, included in the study, completing follow-up, and analysed | 8 | Two hundred and fifty patients were diagnosed with melioidosis and managed by the RDH Infectious Diseases Department between 1^st^ October 2009 and 30^th^ September 2012. Twenty-seven (10.8%) of these died during the intensive phase, one was a relapse of a case diagnosed prior to 1^st^ October 2009 and a further seven had incomplete antibiotic data due to missing files or partially interstate treatment; these patients were excluded leaving 215 patients in the study. All 215 patients were followed up as outlined in the methods and data analysis was performed on all patients except where stated. |
|  |  | (b) Give reasons for non-participation at each stage |  | As outlined in 13 (a). |
|  |  | (c) Consider use of a flow diagram |  | Not required as the above information is sufficiently clear and succinct. |
| Descriptive data | 14* | (a) Give characteristics of study participants (eg demographic, clinical, social) and information on exposures and potential confounders | 9  27 | Baseline characteristics are shown in Table 2. The median (IQR) age was 49.6 (39.0-60.5) years; 119 (55.3%) patients were male and 15 (7.0%) were under 18 years of age. The cohort had a high rate of comorbidity with 181 (84.2%) patients having at least one recognized risk factor for melioidosis. In addition, melioidosis was of characteristic severity with 128 (59.5%) patients bacteremic, 47 (21.9%) requiring intensive care and 32 (14.9%) developing septic shock.  Table 2. |
|  |  | (b) Indicate number of participants with missing data for each variable of interest | 28  32 | Footnote: There were no missing values.  Footnote: There were no missing values. |
|  |  | (c) *Cohort study*—Summarise follow-up time (eg, average and total amount) | 10 | The median (range) duration of follow-up from the onset of the eradication phase for these cured patients was 45.9 (28.4 – 61.1) months. |
| Outcome data | 15* | *Cohort study*—Report numbers of outcome events or summary measures over time | 29  9  9  10  10  10-11 | Table 3.  Twenty (9.3%) patients self-discharged during the intensive phase. Of 133 (61.9%) patients who completed their intravenous therapy through the RDH Hospital in the Home program, the median (IQR) duration of infusor therapy was 14 (8-22) days.  Fig. 1  Eradication phase duration was bimodal with one peak occurring at 90 days (93 [43.3%] patients) and a second peak at 0 days (58 [27.0%] patients). Only 108 (50.2%) patients completed the guideline-specified eradication phase. In total, 70.7% of eradication therapy days were with TMP-SMX; 29.3% were with doxycycline. No patients received amoxicillin-clavulanate.  Fig. 3  Of the 215 patients, 197 (91.6%) patients were cured…. Six (2.8%) patients died during the eradication phase… Eleven (5.1%) patients recrudesced after their initial admission… Only one (0.5%) relapse occurred… One (0.5%) patient had reinfection. |
| Main results | 16 | (*a*) Give unadjusted estimates and, if applicable, confounder-adjusted estimates and their precision (eg, 95% confidence interval). Make clear which confounders were adjusted for and why they were included | 31  12 | As for 15 above. Also:  Table 4  The median (IQR) age for patients who did and did not recrudesce was 46.3 (41.2 to 55.6) and 49.6 (38.8 – 59.9) years respectively (p = 0.47). The only variables significantly predicting recrudescence were diabetes, having osteomyelitis as an ADDF, admission to ICU, septic shock and self-discharge. Multivariate analysis showed that only two of these variables were statistically significant independent predictors of recrudescence; these were self-discharge (odds ratio 6.2 [95% confidence interval 1.2-30.0, p < 0.05]) and septic shock (odds ratio 5.3 [95% confidence interval 1.1-25.7, p < 0.05]). |
|  |  | (*b*) Report category boundaries when continuous variables were categorized |  | N/A |
|  |  | (*c*) If relevant, consider translating estimates of relative risk into absolute risk for a meaningful time period |  | N/A |

Continued on next page

| Other analyses | 17 | Report other analyses done—eg analyses of subgroups and interactions, and sensitivity analyses | 12 | A subgroup analysis was performed on the 58 patients who took no eradication therapy; 52 (89.7%) were cured, 2 (3.4%) died during the eradication phase, 3 (5.2%) recrudesced and 1 (1.7%) relapsed. |
| --- | --- | --- | --- | --- |
| Discussion | | | | |
| Key results | 18 | Summarise key results with reference to study objectives | 14  14  15 | The current guideline is associated with a median 26 day intensive phase duration. That only 1/215 (0.5%) patients relapsed and yet only 50.2% of patients completed the eradication phase suggests that the longer duration of the intensive phase is important in preventing relapse. It is possible that further relapses could occur after the follow-up period but, considering that our shortest follow-up duration, 28.4 months, well exceeds the median time to relapse in the NT, 8 months [[4](#_ENREF_4)], most relapses would be expected to occur within the follow-up period.  That 42 of 43 (97.7%) cases where clinicians were not adherent to the local guideline were cured suggests that the guideline can be further refined...  Whilst the recrudescence rate was higher than desired at 5.1%, there are no previous data, including from our region, on strictly-defined recrudescence with which to compare our current data. |
| Limitations | 19 | Discuss limitations of the study, taking into account sources of potential bias or imprecision. Discuss both direction and magnitude of any potential bias | 16  16-17 | One limitation is that much follow-up data was collected retrospectively…. We acknowledge that there remains a theoretical possibility that patients with recrudescent or recurrent melioidosis diagnosed after migration overseas may have been missed.  Another limitation is that results are compared with historical findings [[4](#_ENREF_4)] and attribution of improvement in relapse rate to the guideline may be confounded by other interventions… There has been a significant improvement in the melioidosis mortality rate over the last twenty years in our region, attributed largely to improved intensive care [[4](#_ENREF_4)], which has meant that more patients with septic shock are surviving; these patients are at higher risk of recrudescence and relapse [[5](#_ENREF_5)] and yet they are not relapsing. |
| Interpretation | 20 | Give a cautious overall interpretation of results considering objectives, limitations, multiplicity of analyses, results from similar studies, and other relevant evidence | 17 | Our findings have important implications for management of melioidosis globally. Relapse rates with traditional approaches to melioidosis therapy are high and present a significant risk to patients and a burden on healthcare resources. Adoption of our guidelines may significantly reduce relapse rates... Given the near-zero relapse rate in our study, despite poor adherence to eradication therapy, further research is required to evaluate eradication phase duration and necessity for different ADDFs following guideline-concordant therapy. If abbreviating or ceasing therapy after the intensive phase were associated with no excess relapses, this would significantly shorten and improve tolerability and safety of the overall treatment regimen and obviate the need for long term follow up, thus further improving the cost-benefit ratio of our approach. |
| Generalisability | 21 | Discuss the generalisability (external validity) of the study results | 16  16-17 | The strengths of our study are the number of patients included and that most data were collected prospectively… the NT has a robust shared electronic health record facilitated by an NT-wide unique patient identification number allowing confirmation of outcome. Additionally, as melioidosis is a notifiable disease in the NT, all Australian laboratories are mandated to report positive cultures of NT patients to the NT public health unit which liaises with the RDH Infectious Diseases Department…  Nonetheless, we are not aware of any other changes in management which may have confounded outcomes. There is biological plausibility in attributing lower relapse rates to the guideline in that intensive therapy uses potent bactericidal antibiotics whereas eradication therapy uses bacteriostatic antibiotics. There has been a significant improvement in the melioidosis mortality rate over the last twenty years in our region, attributed largely to improved intensive care [[4](#_ENREF_4)], which has meant that more patients with septic shock are surviving; these patients are at higher risk of recrudescence and relapse [[5](#_ENREF_5)] and yet they are not relapsing. Based on the above data and decades of clinical experience, we do not believe that there is equipoise in our region for a randomized controlled trial comparing traditional two weeks’ intensive therapy with our guideline minimum intensive phase duration. |
| Other information | |  | | |
| Funding | 22 | Give the source of funding and the role of the funders for the present study and, if applicable, for the original study on which the present article is based |  | Included in submission information: This study was supported in part by the Australian National Health and Medical Research Council through Project Grants, numbers 605820 and 1046812, awarded to BJC. The funder had no role in study design, data collection or analysis, decision to publish or preparation of the manuscript. |

*Give information separately for cases and controls in case-control studies and, if applicable, for exposed and unexposed groups in cohort and cross-sectional studies.

**Note:** An Explanation and Elaboration article discusses each checklist item and gives methodological background and published examples of transparent reporting. The STROBE checklist is best used in conjunction with this article (freely available on the Web sites of PLoS Medicine at http://www.plosmedicine.org/, Annals of Internal Medicine at http://www.annals.org/, and Epidemiology at http://www.epidem.com/). Information on the STROBE Initiative is available at www.strobe-statement.org.
